# White Matter Features Associated With Autistic Traits in Obsessive-Compulsive Disorder

**DOI:** 10.3389/fpsyt.2018.00216

**Published:** 2018-05-29

**Authors:** Masaru Kuno, Yoshiyuki Hirano, Akiko Nakagawa, Kenichi Asano, Fumiyo Oshima, Sawako Nagaoka, Koji Matsumoto, Yoshitada Masuda, Masaomi Iyo, Eiji Shimizu

**Affiliations:** ^1^Research Center for Child Mental Development, Chiba University, Chiba, Japan; ^2^Department of Radiology, Chiba University Hospital, Chiba, Japan; ^3^Department of Psychiatry, Graduate School of Medicine, Chiba University, Chiba, Japan; ^4^Department of Cognitive Behavioral Physiology, Graduate School of Medicine, Chiba University, Chiba, Japan

**Keywords:** obsessive-compulsive disorder, diffusion tensor imaging, white matter tracts, autism spectrum disorder, Autism-Spectrum Quotient, left uncinate fasciculus

## Abstract

Obsessive-compulsive disorder (OCD) is among the most debilitating psychiatric disorders. Comorbid autism spectrum disorder (ASD) or autistic traits may impair treatment response in OCD. To identify possible neurostructural deficits underlying autistic traits, we performed white matter tractography on diffusion tensor images (DTI) and assessed autistic trait severity using the Autism-Spectrum Quotient (AQ) in 33 OCD patients. Correlations between AQ and the DTI parameters, fractional anisotropy (FA), mean diffusivity (MD), axial diffusivity (AD), and radial diffusivity (RD) were examined in major white matter tracts that were suggested to be altered in previous OCD studies. We found a negative correlation between AQ and FA and positive correlations between AQ and MD, AD and RD in the left uncinate fasciculus using age, Beck Depression Inventory, Yale-Brown Obsessive-Compulsive Scale, intelligence quotient and medication as covariates. However, we could not detect the significant results between AQ and all DTI parameters when adding gender as a covariate. In addition, in the ASD comorbid group, FA in the left uncinate fasciculus was significantly lower than in the non-ASD comorbid group and MD and RD were significantly higher than in the non-ASD group. These results did not survive correction for multiple comparisons. In ASD, the socio-emotional dysfunction is suggested to be related to the alteration of white matter microstructure in uncinate fasciculus. Our results suggest that variations in white matter features of the left uncinate fasciculus might be partly explained by autistic traits encountered in OCD patients.

## Introduction

Obsessive-compulsive disorder (OCD) is the fourth most common psychiatric disorder (1), with an approximate lifetime prevalence of 1–3% (2), and is ranked as one of the most debilitating disorders by the World Health Organization (3). It is characterized by both obsessions, defined as recurrent thoughts, urges, or images experienced as intrusive and unwanted and compulsions, repetitive behaviors, or mental acts executed for preventing anxiety or some feared outcome (4).

According to the National Institute for Health and Care Excellence (NICE) treatment guideline, adults with OCD should be offered cognitive behavior therapy (CBT), selective serotonin reuptake inhibitor (SSRI) therapy, or combined SSRI/CBT treatment. However, OCD appears to have a low remission rate and a high probability of recurrence. In a 3-year perspective follow-up of SSRI-treated OCD patients, only 65% achieved partial remission and the relapse probability was 60% (5). In a 15-year follow-up, OCD remission probability was 42% and the recurrence rate was 25% at year 15 (6). Psychiatric comorbidity is strongly associated with poor treatment response (5, 6), including comorbid autism spectrum disorder (ASD) (7). Bejerot et al. reported that approximately 20% of OCD patients have ASD traits. However, in general, these traits were not considered in treatments because these traits were regarded as personality disorders or OCD symptoms (8). Thus, ASD traits were overlooked, which may have contributed to OCD treatment resistance.

ASD is characterized by persistent deficits in social communication and interaction across multiple contexts, as well as restricted, repetitive patterns of behavior, interests, and activities that usually emerge during early development (4). Estimated worldwide prevalence is approximately 1%, and 70% have other psychiatric comorbidities, most frequently anxiety (42–56%), depression (12–70%), and OCD (7–24%) (9).

These high rates of psychiatric comorbidity in ASD may be due to shared etiology, the social disadvantages of autism, or overlapping diagnostic criteria (9). Jacob et al. reviewed overlaps in clinical phenotypes, familial relationships, and genetics between ASD and OCD (10). The shared clinical phenotypes of ASD and OCD include repetitive patterns of behavior, interests, and activities, while obsessive-compulsive (OC) symptoms common to ASD include symmetry obsessions and repeating, ordering, and counting compulsions. On the other hand, aggression, sexual, religious, and somatic obsessions, and checking compulsions or cleaning and contamination are infrequent in ASD (11). Bolton et al. observed that OCD was common in autistic probands and that relatives of OCD patients were more likely to exhibit autistic-like social and communication impairments (12). Hollander et al. found that parents of ASD children exhibiting high scores on the repetitive behavior domain of the Autism Diagnostic Interview-Revised (ADI-R) (13) had more frequent OC traits than parents of ASD children with low ADI-R repetitive behavior domain scores (14). In a prospective study of 3,380,170 cohort samples, ASD patients had a two-fold higher risk of later OCD diagnosis, whereas OCD patients had an almost four-fold higher risk of later ASD diagnosis, strongly suggesting overlapping etiology (15). Moreover, genetic studies have linked chromosome7q (16), chromosomes 1, 6, and 19 (17), and chromosome15q11–13 (18) to OC symptoms in ASD. Serotonin transporter (SERT, SLC6A4), tryptophan hydroxylase (TH2), and 5-HT2B were identified as candidate genes in both OCD and ASD (10). The glutamine transporter gene SLC1A1 and a glutamate receptor gene (GRIK2 or GLUR6) have also been implicated in both OCD and ASD (10).

Previous findings of neuroimaging studies have been reported in both OCD and ASD. In OCD, functional and structural neuroimaging studies suggest that there are abnormalities in the orbitofrontal cortex (OFC), anterior cingulate cortex (ACC), thalamus, and striatum, and the cortico–striato–thalamo–cortical (CSTC) circuit's dysfunction is central (19). Menzies et al. proposed a revised CSTC-based OCD model involving the dorsolateral prefrontal cortex, anterior cingulate, and amygdala (20). In a meta-analysis (21), widespread structural abnormalities in the dorsomedial, dorsolateral, ventrolateral, and frontopolar prefrontal cortices and related areas (temporo-parieto-occipital–associated regions) were found.

In addition to functional neuroimaging studies, diffusion tensor imaging (DTI) studies have suggested white matter (WM) alterations in OCD. DTI measures WM microstructure by quantification of the directionality and coherence of water diffusion. Tissues with highly regular fiber orientation have high fractional anisotropy (FA), while those with less regular fibers have low FA (22). FA is the most commonly reported parameter in DTI studies. Mean diffusivity (MD) is the average of three eigenvalues and is related to WM maturation (23), whereas axial diffusivity (AD) and radial diffusivity (RD) are specific indices with directional eigenvalues (24, 25). Although the exact structural and functional correlates of these parameters remain unclear, they clearly indicate microstructural changes in WM tracts. In previous OCD studies, WM abnormalities were reported in areas including the cingulate bundle, corpus callosum, and anterior limb of the internal capsule but findings were inconsistent (26, 27), possibly due to differences in DTI methodology and OC symptoms heterogeneity. Piras et al. (21) reported consistent alterations of WM in the fronto-basal pathways associated with the OFC and the anterior cingulate cortex. They also reported that the connectivity between the lateral frontal and parietal regions, and microstructural abnormalities in intra-hemispheric bundles have consistently changed (27).

In the volumetric studies to evaluate macrostructure in WM tracts, increased WM-volume was also reported in arcuate and uncinate fasciculus (UF) (28). On the other hand, previous DTI studies have also identified WM microstructure abnormalities in ASD. Travers et al. (29) reported that young adults with ASD tend to have decreased FA accompanied by increased MD and RD in many brain regions, especially in corpus callosum, cingulum, and WM tracts connecting subregions of the temporal lobe (29). In addition to abnormalities of specific brain regions, atypical neural network connectivity such as lower connectivity in the fronto-posterior region in fMRI (functional magnetic resonance imaging) and DTI studies was also suggested (30, 31). Aoki et al. (32) reported the long-distance underconnectivity hypothesis of ASD with significant FA reductions in left uncinate fasciculus in their meta-analysis (32). However, these results were heterogeneous especially in DTI studies (29).

**Figure 1:**
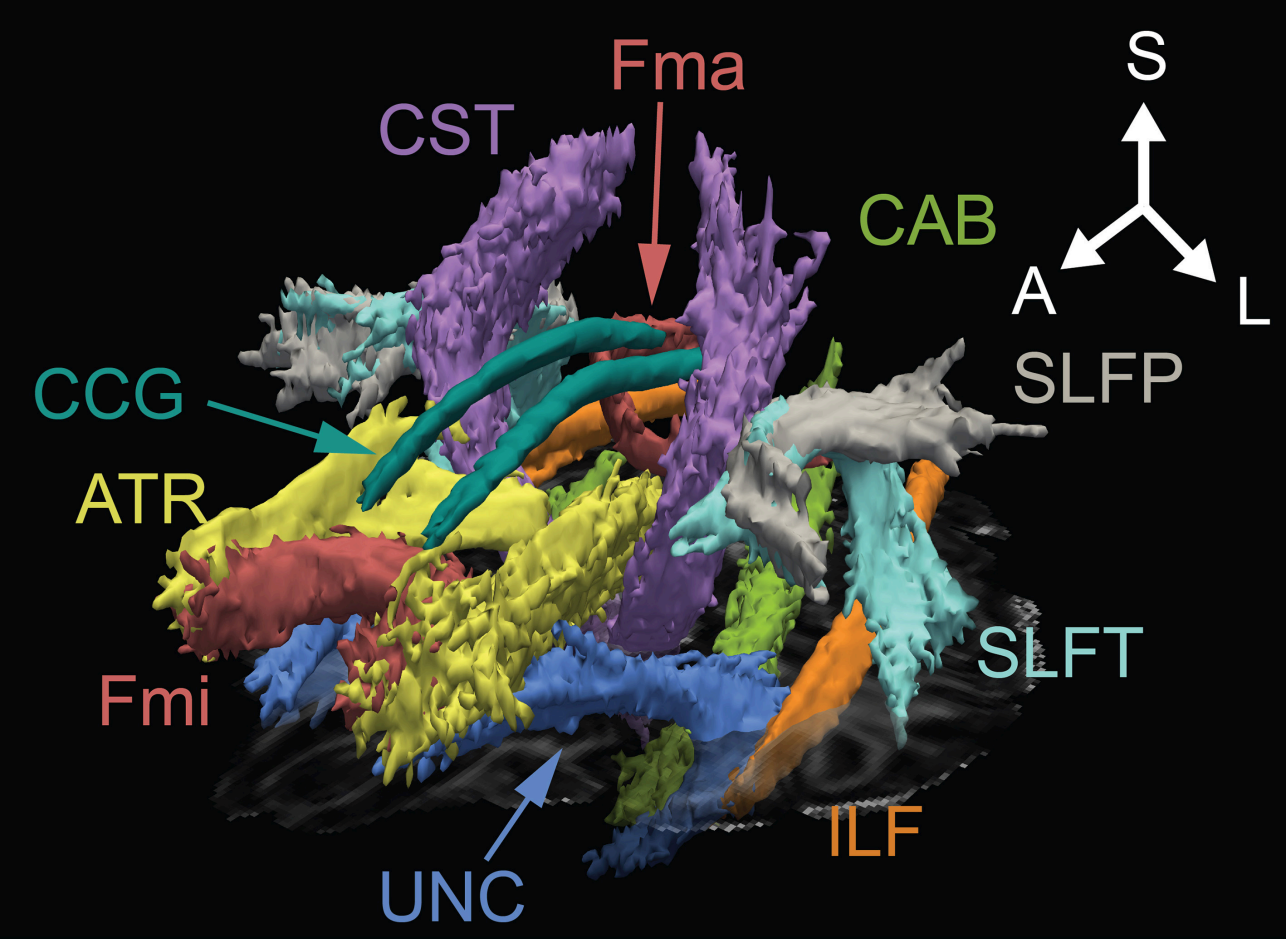
Three-dimensional anatomical representation of reconstructed white matter in one OCD patient. A, anterior; ATR, anterior thalamic radiation; CAB, cingulum angular bundle; CCG, cingulum cingulate gyrus; CST, corticospinal tract; Fma, forceps major; Fmi, forceps minor; ILF, inferior longitudinal fasciculus; L, left; S, superior; SLFP, superior longitudinal fasciculus-parietal; SLFT, superior longitudinal fasciculus-temporal; UNC, uncinate fasciculus.

These relationships between OCD and ASD were also examined in several neuroimaging studies. Ecker et al. (33) reported that gray matter volume of the left dorsolateral prefrontal volume is correlated with the severity of ADI-R repetitive domain symptoms in adult patients with ASD. This area is part of the cognitive control network, and the regional overlap with the revised CSTC circuitry mediating repetitive behaviors may be related to OCD symptoms (33). Kobayashi et al. (35) reported correlations between the Autism-Spectrum Quotient (AQ) (34) scores and regional gray matter volumes in the left dorsolateral prefrontal cortex and amygdala of OCD patients (35). Carlisi et al. (36) reported that the rostrodorsomedial prefrontal cortex in both ASD and OCD showed lower function and structure by their meta-analysis of fMRI and structural MRI (36). WM alterations were investigated in children with neurodevelopmental disorders, such as ASD, OCD, and attention deficit hyperactivity disorder (ADHD) (37). They also reported lower FA within the splenium of the corpus callosum WM alterations in all neurodevelopmental disorders, such as ASD, OCD, and ADHD, than in the control group. However, the studies that investigated both ASD and OCD, and studies that investigated OCD comorbid with ASD, or studies that intended to elucidate how ASD traits in OCD involved in neuropathology of OCD were quite few. The aim of this study was to investigate relationships between regional WM features and comorbid ASD traits in OCD using DTI and the Japanese version of AQ (38, 39). A previous meta-analysis showed widespread WM alterations of OCD (27, 40), so 18 major WM tracts were investigated (Figure 1).

## Methods

### Subjects

Thirty-three patients (12 males and 21 females, 18–48 years) from the Chiba University Psychiatric Imaging Database were enrolled as subjects. All were outpatients of Chiba University Hospital. Diagnosis of OCD was confirmed by trained psychiatrists according to the psychosis subsections of the Structured Clinical Interview for DSM-IV Axis I Disorders, Research Version, Patient Edition (SCID-I/P) (41). We assessed autistic traits by the AQ, OCD severity using Yale-Brown Obsessive-Compulsive Scale (Y-BOCS) (42), and depressive symptoms using Beck Depression Inventory (BDI) (43). Patients with Y-BOCS scores < 16 were excluded. Intelligence was assessed by Wechsler Adult Intelligence Scale (WAIS-III) (44), and patients with intelligence quotient (IQ) < 80 were excluded. We used the Mini-International Neuropsychiatric Interview (M.I.N.I.) to assess comorbidities (45). Handedness was determined by the Edinburgh Handedness Inventory (46). Exclusion criteria were neurological disorders, schizophrenia, substance dependence, organic brain disease, and severe physical diseases. The mean duration of illness was 10.4 ± 7.7 years, the mean total Y-BOCS score was 26.2 ± 3.5, and the mean total AQ score was 25.1 ± 6.8. The number of patients with/without medication was 24 (73%)/9 (27%). There were six patients with medication with both SSRIs and major tranquilizers, 13 taking SSRI, four taking tranquilizers, and one taking clomipramine, a tricyclic antidepressant. In 33 OCD patients, three had comorbidities with ASD and MDD (major depressive disorder), one had comorbidity with ASD and SAD (social anxiety disorder), and one each had comorbidity with MDD, bulimia, agoraphobia, and GAD (generalized anxiety disorder). Nine had comorbidity with only ASD, one had comorbidity with only MDD, and one had comorbidity with only SAD. The number of patients with/without comorbidity was 16 (48%)/17 (52%). Clinical diagnoses of ASD were confirmed with Diagnostic and Statistical Manual of Mental Disorders (DSM)-IV by two experienced psychiatrists, and thirteen patients were diagnosed as having ASD as comorbidity. Detailed clinical and demographic data for all participants are shown in Table 1, and detailed clinical and demographic data for participants comorbid ASD or Non-ASD group are shown in Table 1. The Institutional Research and Ethics Committee of the Graduate School of Medicine, Chiba University approved the study (No. 1330). Written informed consent was obtained from each subject before the assessments began. All experiments were performed in accordance with the Helsinki Declaration. The trial was registered as UMIN000008765.

**Table 1:** Demographic, clinical data, and head motion of patients with OCD.

**Variable**	**Patients**		**ASD**		**Non-ASD**			
	***N* (%)**	**Mean (SD)**	**Range**	***N* (%)**	**Mean (SD)**	***N* (%)**	**Mean (SD)**	***P*-value**
Age (years)		32.8 (7.7)	18–48		31.5 (8.2)		33.7 (7.5)	0.298
Gender (male/female)	12/21			9/4		5/15		0.029^*^^†^
Handedness (right/left)	33/0			13/0		20/0		–
Age of onset of OCD (years)		22.4 (8.1)	6–40		20.9 (6.5)		23.4 (9.1)	0.465
Duration of illness (years)		10.4 (7.7)	0–25		10.8 (7.7)		10.1 (7.9)	0.917
Y-BOCS		26.2 (3.5)	19–34		27.1 (3.1)		25.6 (3.7)	0.372
AQ		25.1 (6.8)	10–39		28.5 (4.8)		23.0 (7.1)	0.005^*^
BDI		18.2 (11.0)	2–44		23.9 (6.2)		15.1 (12.0)	0.011^*^
FIQ		102.2 (11.4)	80–122		103.2 (9.9)		101.5 (12.5)	0.624
Comorbidities								
Major depressive disorder	4 (12)			3 (23)		3 (15)		0.659^†^
Social anxiety disorder	2 (6)			1 (8)		1 (5)		1.000^†^
Generalized anxiety disorder	1 (3)			0		1 (5)		–
Bulimia	1 (3)			0		1 (5)		–
Agoraphobia	1 (3)			0		1 (5)		–
Medication at time of study								
Medication-free	9 (27)			4 (31)		5 (25)		1.000^†^
SSRIs	13 (39)			4 (31)		9 (45)		0.485^†^
Antipsychotic augmentations	6 (18)			4 (31)		2 (10)		0.182^†^
Major tranquilizers	4 (12)			0		4 (20)		–
Clomipramine	1 (3)			2 (15)		0		–
Head motion								
Translation (mm)		0.773 (0.261)			0.880 (0.288)		0.563 (0.115)	0.057
Rotation (degrees)		0.005 (0.003)			0.006 (0.003)		0.004 (0.001)	0.049^*^

SD, standard deviation; OCD, obsessive-compulsive disorder; Y-BOCS, Yale-Brown Obsessive-Compulsive Scale; AQ, Autism Spectrum Quotient; BDI, Beck Depression Inventory; FIQ, Full scale Intelligence Quotient assessed by WAIS-III, SSRIs, selective serotonin reuptake inhibitors; SSRIs, number of patients taking only SSRIs; Antipsychotic augmentations, number of patients taking both SSRIs and major tranquilizers; Major tranquilizer, number of patients taking only major tranquilizers; Mean of chlorpromazine equivalent of major tranquilizers for each patient (92.7 ± 65.6 mg). Mean of imipramine equivalent of antidepressants for each patient (138.4 ± 65.1 mg).

* p < 0.05,

† *Fisher's exact test*.

### MRI acquisition

All subjects underwent DTI and T1-weighted MRI using a 3T scanner equipped with a 32-channel phased-array head coil (Discovery MR750 3.0 T; GE Healthcare, Waukesha, WI, USA). Images were collected by 3D fast spoiled gradient-echo (FSPGR) sequence (echo time: 3.164 ms; repetition time: 8.124 ms; flip angle: 15°; acquisition matrix: 256 × 256; slice thickness: 1 mm; field of view: 25.6 × 25.6 cm^2^; number of excitations: 1; bandwidth: 31.25 kHz; inversion time: 420 ms; acceleration factor: 2). DTI data were acquired using a single-shot echo-planar sequence with the following parameters: TE = 61.1 ms, TR = 8,500 ms, matrix size = 128 × 128, imaging resolution = 1.875 × 1.875 × 2 mm^3^, band width = 250 kHz, number of motion-probing gradient (MPG) directions = 30, *b*-value = 1,000 s/mm^2^, number of acquisitions = 2, acceleration factor = 2.

### MRI data processing

DTI and T1-weighted images of each subject were processed in FreeSurfer 5.3.0 (https://surfer.nmr.mgh.harvard.edu) (47) using the TRACULA (TRActs Constrained by UnderLying Anatomy) toolbox (48) with a probabilistic tractography according to a method reported in a previous study (49). In image processing, head motions and eddy currents were corrected and image quality was checked (50). Subsequently, intra-subject registration was performed by affine transformations between low-b diffusion and structural T1-weighted images. Then, the individual's T1 images and DTI images were registered to MNI template (51). In addition to a cortex mask, a WM mask was created by cortical parcellation and subcortical segmentation transformed to the template image. Then, all scalar diffusion measures (FA, MD, AD, and RD) were mapped to the template image after fitting by least-squares tensor estimation using DTI fit function in the Oxford Center for Functional MRI of the Brain (FMRIB) Software Library (FSL) 5.0 (www.fmrib.ox.ac.uk/fsl/), and a priori probabilities of WM pathways were computed and mapped the selected initial control points from the template space to individual diffusion space. Moreover, fitting of the ball-and-stick model of diffusion (52) was performed to estimate the posteriori probability distribution of 18 major WM pathway's (53) location in the individual and tensor-scalar values were extracted for each pathway (54).

### Statistical analysis

All statistical calculations were performed using SPSS software (version 21.0, IBM Corp., Armonk, NY). The difference of clinical and demographic data between ASD and Non-ASD group were investigated using Mann-Whitney *U*-tests or Fisher's exact test. Correlations between AQ scores and DTI parameters (FA, MD, AD, and RD) were investigated in target WM tracts (the corpus callosum forceps major, corpus callosum forceps minor, bilateral anterior thalamic radiation, bundle of cingulum, cingulate gyrus of cingulum, corticospinal tract, inferior longitudinal fasciculus, parietal part of the superior longitudinal fasciculus, temporal part of superior longitudinal fasciculus, and UF). Head motions during DTI scans were evaluated by two-sample *t*-test after test of normality in average translation and average rotation, percent of bad slices, and drop out scores. The partial correlation coefficient was obtained using age, BDI, Y-BOCS, FIQ, medication, and gender as control variables. The differences of WM features were investigated between OCD patients divided into two groups according to ASD diagnosis using Mann-Whitney *U*-tests. The significance level was set at *p* < 0.05 (two-sided). Y-BOCS total score were used as a nuisance covariate in order to adjust for the effect of OCD severity in WM features.

## Results

Average translation and average rotation in head motion in with or without ASD group were 0.880 ± 0.288 mm and 0.006 ± 0.003 degrees and 0.563 ± 0.115 mm and 0.004 ± 0.001) degrees, respectively. No significant difference in head translation (*p* = 0.057) was shown but the larger head rotation was shown in OCD comorbid with ASD group (*p* = 0.049). No percent of bad slices were shown in both groups. Drop out scores were one in both groups (Table 1).

Partial correlations were investigated using age, BDI, Y-BOCS, FIQ, and medication as covariates. A significant negative correlation was observed between AQ score and FA in the left UF (*r* = −0.47, *p* = 0.015, *d* = 1.06) and FA in the left cingulum angular bundle (*r* = −0.43, *p* = 0.034, *d* = 0.94), and significant positive correlations were observed between AQ score and MD, AD, and RD in the left UF (MD, *r* = 0.49, *p* = 0.012, *d* = 1.12; AD, *r* = 0.47, *p* = 0.016, *d* = 1.06; and RD, *r* = 0.49, *p* = 0.011, *d* = 1.12), MD in the right cingulum cingulate gyrus (*r* = 0.41, *p* = 0.048, *d* = 0.89), AD in the right cingulum angular bundle (*r* = 0.46, *p* = 0.024, *d* = 1.04). However, correlation analysis did not survive the multiple comparisons (Holms method). We also added gender as a covariate because gender should be adapted to control variables for their effect on WM integrity (55). Then, we could not detect the significant results between AQ and DTI parameters in all 18 pathways. There were three outliers in DTI parameters. They were all male with the high AQ scores (35) and average age (mean; 33.0, range; 27–37), Y-BOCS (mean; 26.3, range: 26–27), and BDI (mean; 20.7, range 15–28). After deleting these three individuals, significant correlations were not shown.

The differences of WM features in the left UF were investigated between OCD patients divided into two groups according to ASD diagnosis using Mann-Whitney *U*-test. In the ASD comorbid group, FA (*t* = −2.58, *p* = 0.015, *r* = 0.42, *d* = 0.93) was significantly lower, and MD (*t* = 2.14, *p* = 0.040, *r* = 0.36, *d* = 0.77) and RD (*t* = 2.27, *p* = 0.030, *r* = 0.38, *d* = 0.82) were significantly higher than in the non-ASD comorbid group in the left UF. These results could clarify that the ASD traits in OCD might affect WM features in this pathway.

## Discussion

**Figure 2:**
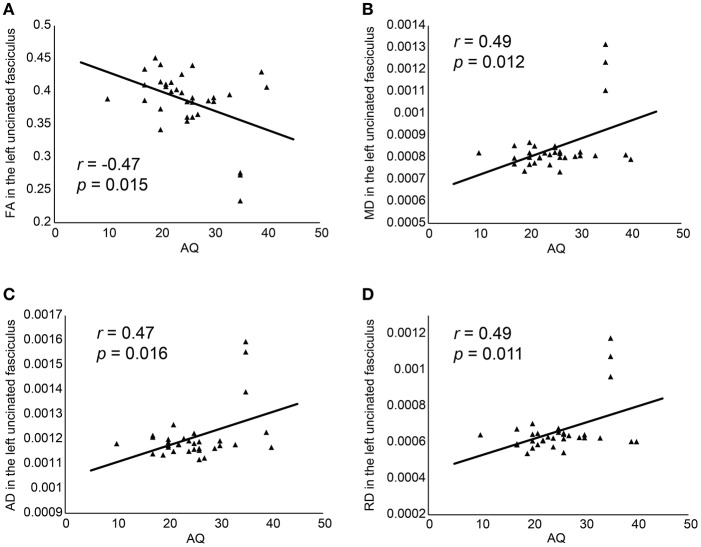
Correlation between AQ and FA **(A)**, MD **(B)**, AD **(C)**, and RD **(D)**. AD, axial diffusivity; AQ, Autism-Spectrum Quotient; FA, fractional anisotropy; MD, mean diffusivity; RD, radial diffusivity.

We observed a negative correlation between AQ scores and FA and positive correlations between AQ scores and MD, AD, and RD in the left UF, FA in left cingulum angular bundle, MD in right cingulum cingulate gyrus, AD in right cingulum angular bundle using age, BDI score, Y-BOCS, medication and FIQ as covariates (Figure 2), and significant differences in FA, MD, RD when we compared the WM integration in the left UF according to the ASD diagnosis of patient. Functional interpretations are difficult because individual parameters are intricately interrelated with all others. For example, in WM neuropathology, the anisotropy decreases when either RD increases and/or RD reduces (24). However, previous DTI studies of ASD showed reduced FA and elevated MD and RD (56), and this correlation pattern was shown in the current study. These results suggest that the degree of WM feature in the left UF is associated with the severity of autistic traits in OCD. In the current study, we did not detect any significant results when adding gender as a covariate in addition to BDI score, Y-BOCS, medication, and FIQ. A voxel wise study reported the differences of FA, RD, and AD between men and women in many brain regions such as splenium of the corpus callosum, bilateral corona radiate, posterior limb of the internal capsule, cerebral peduncle, external capsule, bilateral superior longitudinal fasciculus, bilateral middle cerebellar peduncle, and the column of the fornix (55). Hence, it was not denied that the possibility of the existence of gender effects in the current results.

Von Der Heide et al. (57) proposed three major functions of UF, including associative and episodic memory functions, linguistic functions, and social-emotional functions (57). One of the reasons they proposed socio-emotional functions was an anatomical reason is that UF connects limbic circuit involved in social memories as well as theory of mind to OFC that is considered the center of reward-based decision making. Another reason is that clinical populations with socio-emotional dysfunction, such as fronto-temporal dementia (58–60), traumatic brain injury (61, 62), Capgras delusion (63, 64) and so on, represented damages to UF. Then, they summarized the function of UF as the intersection of memory and social-emotional processes that associated the temporal lobe function involved in integrating a person's name, face, voice, and feeling for a person, and the orbito-frontal function involved in reward-based decision making. On the other hand, in OCD, Menzies et al. proposed a revised CSTC loop that included the limbic region associated with affective impairment in OCD from functional and structural MRI findings (20). In an fMRI study of OCD, the limbic, temporal, and hypothalamus regions were activated when a shame-related task was presented, and the frontal, limbic, and temporal parts were activated when a guilt-related task was presented (65). Decreased FA in the right UF, positive correlation between FA in the cingulum/UF and symptom severity (66), increased MD in the left UF (67) were also reported. Jayarajan et al. (68) reported WM alterations in UF and suggested the possibility of the limbic system involvement that played a role in emotional processing (68). Zarei et al. (69) indicated the high FA of right UF and the correlation of left UF with symptom severity in OCD patients (69). However, the effect of autistic traits on white matter alterations in UF were not considered in these previous OCD studies. Then, the correlation pattern in the current study might indicate the effect of highly autistic traits on WM integrity in OCD patients.

In previous studies of ASD, UF is associated with socio-emotional functioning (56). Normal infants gradually develop abilities for discrimination of emotional expressions in others, joint attention, and imitative behaviors (70), with a comprehensive theory of mind established by age 4 years (70). On the other hand, infants with ASD exhibit less interest in social stimuli, as well as less imitation, eye contact, and social smiling (70). They show little reaction to the facial expressions of others, less social imitation, less joint attention, and an impaired theory of mind (70). These socio-emotional deficits in ASD are associated with limbic and paralimbic systems' dysfunction. The UF plays an important role in socio-emotional function by connecting the temporal lobe, amygdala, and hippocampus to insular and orbitofrontal cortices (56). Structural MRI studies of ASD have revealed widespread abnormalities that include but are not specific to the UF (56). A previous fMRI study of ASD reported a correlation between reduced connectivity among limbic and non-limbic regions and social symptom severity on the Social Responsiveness Scale (71), and this impaired limbic/non-limbic connectivity included UF deficits (56). For instance, an FA abnormality in the UF at 6 months of age predicted impaired joint attention at 9 months of age (72). In another DTI study, the volume and density of the UF were correlated with autistic features, including socio-emotional deficits (73). Further, FA in the UF correlated with therapy duration and clinical outcome as measured by the Child Autism Rating Scale (74). Samson et al. (75) assessed WM alteration in 18 ASD group and typically developed (TD) controls, and investigated the relationship between FA in UF using DTI and socio-affective deficit. They reported that ASD group had lower FA in left UF than TD, and within ASD group, FA was related to socio-affective deficit (75). These results would support the correlation between AQ representing autistic traits and UF features in the current study, but it would be difficult to evaluate whether our subjects had socio-emotional deficits without functional data of socio-emotional function.

Our study has some limitations. First, our sample size may not be large enough to detect less robust correlations between autistic traits and DTI parameters. Second, the majority of OCD patients took medications, but medication may introduce additional behavioral or neurostructural heterogeneity, thereby obscuring other DTI-AQ associations (76). Third, AQ, used to evaluate autistic traits, and BDI, used to evaluate depressive symptoms, are subjective self-administered questionnaires. More objective assessment tools such as the ADI-R, Autism Diagnostic Observation Second Edition (ADOS-2), and the Hamilton Rating Scale for Depression may be more accurate. Fourth, some of the patients were diagnosed with comorbid major depressive disorder, which may also influence AQ and neuroimaging results (77). Fifth, in our study, we had no functional data demonstrating the socio-emotional dysfunction of UF in OCD patients. Therefore, though the WM feature in left UF might be associated with the socio-emotional function, the validity of this relationship was difficult to determine. Sixth, there were no control groups such as a healthy control group or an ASD group. Seventh, we did not detect significant correlations between AQ and DTI parameters when we added gender as covariates. Our result might be affected by gender. Eighth, all the statistically significant results did not survive Holms correction. Then the result of the current study should be considered carefully. Finally, the decrease of FA and increase of RD might come from head motion (50). Findings from DTI in some recent studies for ASD may be confounded by head motion (78). They revealed that effect sizes of group differences between ASD and healthy control in several tracts increased with stringent quality matching. Careful quality-control and motion-matching should be considered. Future studies with larger numbers, less severe or absent comorbidities, controlled medication, objective assessment tools, comparison with a control group, and with socio-emotional measurements, may allow for the detection of a broader range of associations between ASD traits and WM features in OCD.

Despite these limitations, this is the first study to examine the correlations between AQ and the DTI parameters, FA, MD, AD, and RD in major white matter tracts in OCD. We found correlations between autistic trait severity and the extent of left UF features in OCD patients. The AQ was negatively related to FA in left UF, and positively related to MD, AD, and RD in left UF. These results suggest that variations in WM features of the left UF might be partly explained by autistic traits encountered in OCD patients.

## Author contributions

MK, YH, and AN wrote the manuscript. MK and YH conducted the experiments and analyzed the data. AN recruited subjects and conducted assessments. KM acquired the imaging data. KA, FO, SN, YM, and MI arranged the conditions for the experiment, and ES supervised this study. All authors approved the final manuscript.

## Conflict of interest statement

The authors declare that the research was conducted in the absence of any commercial or financial relationships that could be construed as a potential conflict of interest.

## Abbreviations

OCD: obsessive-compulsive disorder
NICE: the National Institute for Health and Care Excellence
CBT: cognitive behavior therapy
SSRI: selective serotonin reuptake inhibitor
ASD: autism spectrum disorder
OC: obsessive-compulsive
ADI-R: Autism Diagnostic Interview-Revised
OFC: orbitofrontal cortex
ACC: anterior cingulate cortex
CSTC: cortico-striato-thalamo-cotrical
DTI: diffusion tensor images
WM: white matter
FA: fractional anisotropy
AD: axial diffusivity
MD: mean diffusivity
RD: radial diffusivity
UF: uncinate fasciculus
MRI: magnetic resonance imaging
fMRI: functional magnetic resonance imaging
AQ: Autism-Spectrum Quotient
ADHD, attention deficit hyperactivity disorder, SCID-I/P, Structured Clinical Interview for DSM-IV Axis I Disorders, Research Version: Patient Edition
Y-BOCS: Yale-Brown Obsessive-Compulsive Scale
BDI: Beck Depression Inventory
WAIS-III: Wechsler Adult Intelligence Scale
IQ: intelligence quotient
M.I.N.I.: Mini-International Neuropsychiatric Interview
MDD: major depressive disorder
SAD: social anxiety disorder
GAD: generalized anxiety disorder
FSPGR: fast spoiled gradient-echo
MPG: motion-probing gradient
TRACULA: TRActs Constrained by UnderLying Anatomy
ADOS-2: Autism Diagnostic Observation Second Edition.

## References

[1] Grant JE. Obsessive–compulsive disorder. N Engl J Med. (2014) 371:646–53. 10.1056/NEJMcp1402176 25119610

[2] Kessler RC Berglund P Demler O Jin R Merikangas KR Walters EE. Lifetime prevalence and age-of-onset distributions of DSM-? disorders in the national comorbidity survey replication. Arch Gen Psychiatry (2005) 62:593–602. 10.1001/archpsyc.62.6.593 15939837

[3] Ayuso-Mateos JL Global Burden of Obsessive-Compulsive Disorder in the Year 2000. Available online at: http://www.whoint/healthinfo/statistics/bod_obsessivecompulsive.pdf. (2000).

[4] American Psychiatric Association Diagnostic and Statistical Manual of Mental Disorders, 5th Edn. Washington, DC: American Psychiatric Publishiing (2013). 992 p.

[5] Catapano F Perris F Masella M Rossano F Cigliano M Magliano L. Obsessive-compulsive disorder: A 3-year prospective follow-up study of patients treated with serotonin reuptake inhibitors. OCD follow-up study. J Psychiatr Res. (2006) 40:502–10. 10.1016/j.jpsychires.2005.04.010 16904424

[6] Marcks BA Weisberg RB Dyck I Keller MB. Longitudinal course of obsessive-compulsive disorder in patients with anxiety disorders: a 15-year prospective follow-up study. Comp Psychiatry (2011) 52:670–7. 10.1016/j.comppsych.2011.01.001 21349511 PMC3683832

[7] Storch EA Björgvinsson T Riemann B Lewin AB Morales MJ Murphy TK. Factors associated with poor response in cognitive-behavioral therapy for pediatric obsessive-compulsive disorder. Bull Menninger Clin. (2010) 74:167–85. 10.1521/bumc.2010.74.2.167 20545494

[8] Bejerot S. An autistic dimension: a proposed subtype of obsessive-compulsive disorder. Autism (2007) 11:101–10. 10.1177/1362361307075699 17353211

[9] Lai MC Lombardo MV Baron-Cohen S. Autism. Lancet (2014) 383:896–910. 10.1016/S0140-6736(13)61539-1 24074734

[10] Jacob S Landeros-Weisenberger A Leckman JF. Autism spectrum and obsessive-compulsive disorders: OC behaviors, phenotypes and genetics. Autism Res. (2009) 2:293–311. 10.1002/aur.108 20029829 PMC3974607

[11] Bloch MH Landeros-Weisenberger A Sen S Dombrowski P Kelmendi B Coric V Association of the serotonin transporter polymorphism and obsessive-compulsive disorder: systematic review. Am J Med Genet Part B Neuropsychiatr Genet. (2008) 147:850–8. 10.1002/ajmg.b.30699 18186076

[12] Bolton PF Pickles A Murphy M Rutter M. Autism, affective and other psychiatric disorders: patterns of familial aggregation. Psychol Med. (1998) 28:385–95. 10.1017/S0033291797006004 9572095

[13] Lord C Rutter M Le Couteur A. Autism diagnostic interview-revised: a revised version of a diagnostic interview for caregivers of individuals with possible pervasive developmental disorders. J Autism Dev Disord. (1994) 24:659–85. 10.1007/BF02172145 7814313

[14] Hollander E King A Delaney K Smith CJ Silverman JM. Obsessive–compulsive behaviors in parents of multiplex autism families. Psychiatry Res. (2003) 117:11–6. 10.1016/S0165-1781(02)00304-9 12581816

[15] Meier SM Petersen L Schendel DE Mattheisen M Mortensen PB Mors O. Obsessive-compulsive disorder and autism spectrum disorders: longitudinal and offspring risk. PLoS ONE (2015) 10:e0141703. 10.1371/journal.pone.0141703 26558765 PMC4641696

[16] Alarcón M Cantor RM Liu J Gilliam TC Geschwind DH. Evidence for a language quantitative trait locus on chromosome 7q in multiplex autism families. Am J Hum Genet. (2002) 70:60–71. 10.1086/338241 11741194 PMC384904

[17] Buxbaum JD Silverman J Keddache M Smith CJ Hollander E Ramoz N. Linkage analysis for autism in a subset families with obsessive-compulsive behaviors: evidence for an autism susceptibility gene on chromosome 1 and further support for susceptibility genes on chromosome 6 and 19. Mol Psychiatry (2004) 9:144–50. 10.1038/sj.mp.4001465 14699429

[18] Shao Y Cuccaro ML Hauser ER Raiford KL Menold MM Wolpert CM. Fine mapping of autistic disorder to chromosome 15q11-q13 by use of phenotypic subtypes. Am J Hum Genet. (2003) 72:539–48. 10.1086/367846 12567325 PMC1180230

[19] Saxena S Brody AL Maidment KM Dunkin JJ Colgan M Alborzian S. Localized orbitofrontal and subcortical metabolic changes and predictors of response to paroxetine treatment in obsessive-compulsive disorder. Neuropsychopharmacology (1999) 21:683–93. 10.1016/S0893-133X(99)00082-2 10633474

[20] Menzies L Chamberlain SR Laird AR Thelen SM Sahakian BJ Bullmore ET. Integrating evidence from neuroimaging and neuropsychological studies of obsessive-compulsive disorder: the orbitofronto-striatal model revisited. Neurosci Biobehav Rev. (2008) 32:525–49. 10.1016/j.neubiorev.2007.09.005 18061263 PMC2889493

[21] Piras F Piras F Chiapponi C Girardi P Caltagirone C Spalletta G. Widespread structural brain changes in OCD : a systematic review of voxel-based morphometry studies. Cortex (2013) 62:89–108. 10.1016/j.cortex.2013.01.016 23582297

[22] Mori S Zhang J. Principles of diffusion tensor imaging and its applications to basic neuroscience research. Neuron (2006) 51:527–39. 10.1016/j.neuron.2006.08.012 16950152

[23] Beaulieu C. The basis of anisotropic water diffusion in the nervous system - A technical review. NMR Biomed. (2002) 15:435–55. 10.1002/nbm.782 12489094

[24] Alexander AL Lee JE Lazar M Field AS. Diffusion tensor imaging of the brain. Neurotherapeutics (2007) 4:316–29. 10.1016/j.nurt.2007.05.011 17599699 PMC2041910

[25] Assaf Y Pasternak O. Diffusion tensor imaging (DTI)-based white matter mapping in brain research: a review. J Mol Neurosci. (2008) 34:51–61. 10.1007/s12031-007-0029-0 18157658

[26] Koch K Reeß TJ Rus OG Zimmer C Zaudig M. Diffusion tensor imaging (DTI) studies in patients with obsessive-compulsive disorder (OCD): a review. J Psychiatr Res. (2014) 54:26–35. 10.1016/j.jpsychires.2014.03.006 24694669

[27] Piras F Piras F Caltagirone C Spalletta G. Brain circuitries of obsessive compulsive disorder: a systematic review and meta-analysis of diffusion tensor imaging studies. Neurosci Biobehav Rev. (2013) 37:2856–77. 10.1016/j.neubiorev.2013.10.008 24177038

[28] Radua J Via E Catani M Mataix-Cols D. Voxel-based meta-analysis of regional white-matter volume differences in autism spectrum disorder versus healthy controls. Psychol Med. (2011) 41:1539–50. 10.1017/S0033291710002187 21078227

[29] Travers B Adluru N Ennis C Tromp D Destiche D Doran S. Diffusion tensor imaging in autism spectrum disorder: a review. Autism Res. (2012) 5:289–313. 10.1002/aur.1243 22786754 PMC3474893

[30] Minshew NJ Keller TA. The nature of brain dysfunction in autism: functional brain imaging studies. Curr Opin Neurobiol. (2010) 23:124–30. 10.1097/WCO.0b013e32833782d4 20154614 PMC2975255

[31] Just MA Kellera TA Malavea VL Kanab RK Varma S. Autism as a neural systems disorder: a theory of frontal-posterior underconnectivity. Neurosci Biobehav Rev. (2012) 36:1292–313. 10.1016/j.neubiorev.2012.02.007 22353426 PMC3341852

[32] Aoki Y Abe O Nippashi Y Yamasue H. Comparison of white matter integrity between autism spectrum disorder subjects and typically developing individuals: a meta-analysis of diffusion tensor imaging tractography studies. Mol Autism. (2013) 4:25. 10.1186/2040-2392-4-25 23876131 PMC3726469

[33] Ecker C Suckling J Deoni SC. Lombardo MV Bullmore ET Baron-Cohen S. Brain Anatomy and Its Relationship to Behavior in Adults With Autism Spectrum Disorder. Arch Gen Psychiatry (2012) 69:195–209. 10.1001/archgenpsychiatry.2011.1251 22310506

[34] Baron-Cohen S Wheelwright S Skinner R Martin J Clubley E. The autism spectrum quotient : evidence from asperger syndrome/high functioning autism, males and females, scientists and mathematicians. J Autism Dev Disord. (2001) 31:5–17. 10.1023/A:1005653411471 11439754

[35] Kobayashi T Hirano Y Nemoto K Sutoh C Ishikawa K Miyata H. Correlation between morphologic changes and autism spectrum tendency in obsessive-compulsive disorder. Magn Reson Med Sci. (2015) 14:329–35 10.2463/mrms.2014-0146 26104070

[36] Carlisi CO Norman LJ Lukito SS Radua J Mataix-Cols D Rubia K. Comparative multimodal meta-analysis of structural and functional brain abnormalities in autism spectrum disorder and obsessive-compulsive disorder. Biol Psychiatry (2017) 82:83–102. 10.1016/j.biopsych.2016.10.006 27887721

[37] Ameis SH Lerch JP Taylor MJ Lee W Viviano JD Pipitone J. A diffusion tensor imaging study in children with ADHD, autism spectrum disorder, OCD, and matched controls: distinct and non-distinct white matter disruption and dimensional brain-behavior relationships. Am J Psychiatry (2016) 173:1213–22. 10.1176/appi.ajp.2016.15111435 27363509

[38] Wakabayashi A Tojo Y Baron-Cohen S Wheelwright S. The Autism-Spectrum Quotient (AQ) Japanese version: evidence from high-functioning clinical group and normal adults. Japn J Psychol. (2004) 75:78–84. 10.4992/jjpsy.75.78 15724518

[39] Wakabayashi A Baron-Cohen S Uchiyama T Yoshida Y Tojo Y Kuroda M. The Autism-Spectrum Quotient (AQ) children's version in Japan: a cross-cultural comparison. J Autism Dev Disord. (2007) 37:491–500. 10.1007/s10803-006-0181-3 16944324

[40] Radua J Grau M van den Heuvel OA Thiebaut de Schotten M Stein DJ Canales-Rodríguez EJ. Multimodal voxel-based meta-analysis of white matter abnormalities in obsessive–compulsive disorder. Neuropsychopharmacology (2014) 39:1547–57. 10.1038/npp.2014.5 24407265 PMC4023155

[41] Michael BF Robert LS Miriam WG Structured Clinical Interview for DSM-IV-TR Axis I Disorders, Research Version. Patient Edition. Biometrics Research, New York State Psychiatric Institute (2002).

[42] Goodman WK Price LH Rasmussen SA Mazure C Fleischmann RL Hill CL. The Yale-Brown Obsessive Compulsive Scale. I. Development, use, and reliability. Archiv Gen Psychiatry (1989) 46:1006–11. 2684084 10.1001/archpsyc.1989.01810110048007

[43] Beck AT Ward CH Mendelson M Mock J Erbaugh J. An inventory for measuring depression. Arch Gen Psychiatry (1961) 4:561–71. 10.1001/archpsyc.1961.01710120031004 13688369

[44] Wechsler D 1981 WAIS-R Manual: Wechsler Adult Intelligence Scale-Revised. New York, NY: Psychological Corporation (1981).

[45] Sheehan DV Lecrubier Y Sheehan KH Amorim P Janavs J Weiller E. The Mini-International Neuropsychiatric Interview (M.I.N.I.): the development and validation of a structured diagnostic psychiatric interview for DSM-IV and ICD-10. J Clin Psychiatry (1998) 59(Suppl. 20):22–33. 9881538

[46] Oldfield RC. The assessment and analysis of handedness: The Edinburgh inventory. Neuropsychologia (1971) 9:97–113. 10.1016/0028-3932(71)90067-4 5146491

[47] Fischl B. FreeSurfer. Neuroimage (2012) 62:774–81. 10.1016/j.neuroimage.2012.01.021 22248573 PMC3685476

[48] Yendiki A Panneck P Srinivasan P Stevens A Zöllei L Augustinack J. Automated probabilistic reconstruction of white-matter pathways in health and disease using an atlas of the underlying anatomy. Front Neuroinform. (2011) 5:23. 10.3389/fninf.2011.00023 22016733 PMC3193073

[49] Won E Kang J Choi S Kim A Han K Yoon H. The association between substance P and white matter integrity in medication-naive patients with major depressive disorder. Sci Rep. (2017) 7:9707. 10.1038/s41598-017-10100-y 28852030 PMC5575350

[50] Yendiki A Koldewyn K Kakunoori S Kanwisher N Fischl B. Spurious group differences due to head motion in a diffusion MRI study. Neuroimage (2013) 88:79–90. 10.1016/j.neuroimage.2013.11.027 24269273 PMC4029882

[51] Collins DL Neelin P Peters TM Evans EA. Automatic 3D intersubject registration of MR volumetric data in standardized Talairach space. J Comput Assist Tomogr. (1994) 18:192–205. 8126267

[52] Behrens TEJ Woolrich MW Jenkinson M Johansen-Berg H Nunes RG Clare S. Characterization and propagation of uncertainty in diffusion-weighted MR imaging. Magn Reson Med. (2003) 50:1077–88. 10.1002/mrm.10609 14587019

[53] Wakana S Caprihan A Panzenboeck MM Fallon JH Perry M Gollub RL. Reproducibility of quantitative tractography methods applied to cerebral white matter. Neuroimage (2007) 36:630–44. 10.1016/j.neuroimage.2007.02.049 17481925 PMC2350213

[54] Behrens TEJ Berg HJ Jbabdi S Rushworth MFS Woolrich MW. Probabilistic diffusion tractography with multiple fibre orientations: what can we gain? Neuroimage (2007) 34:144–55. 10.1016/j.neuroimage.2006.09.018 17070705 PMC7116582

[55] Inano S Takao H Hayashi N Abe O Ohtomo K. Effects of age and gender on white matter integrity. Am J Neuroradiol. (2011) 32:2103–9. 10.3174/ajnr.A2785 21998104 PMC7964377

[56] Ameis SH Catani M. Altered white matter connectivity as a neural substrate for social impairment in Autism Spectrum Disorder. Cortex (2015) 62:158–81. 10.1016/j.cortex.2014.10.014 25433958

[57] Von Der Heide RJ Skipper LM Klobusicky E Olson IR. Dissecting the uncinate fasciculus: disorders, controversies and a hypotheses. Brain (2013) 136:1692–707. 10.1093/brain/awt094 23649697 PMC3673595

[58] Galantucci S Tartaglia MC Wilson SM Henry ML Filippi M Agosta F. White matter damage in primary progressive aphasias: a diffusion tensor tractography study. Brain (2011) 134:3011–29. 10.1093/brain/awr099 21666264 PMC3187537

[59] Acosta-Cabronero J Patterson K Fryer TD Hodges JR Pengas G Williams GB. Atrophy, hypometabolism and white matter abnormalities in semantic dementia tell a coherent story. Brain (2011) 134:2025–35. 10.1093/brain/awr119 21646331

[60] Whitwell JL Avula R Senjem ML Kantarci K Weigand SD Samikoglu A. Gray and white matter water diffusion in the syndromic variants of frontotemporal dementia. Neurology (2010) 74:1279–87. 10.1212/WNL.0b013e3181d9edde 20404309 PMC2860485

[61] Zappalà G Thiebaut de Schotten M Eslinger PJ. Traumatic brain injury and the frontal lobes: What can we gain with diffusion tensor imaging? Cortex (2012) 48:156–65. 10.1016/j.cortex.2011.06.020 21813118

[62] Johnson CP Juranek J Kramer LA Prasad MR Swank PR. Predicting behavioral deficits in pediatric traumatic brain injury through uncinate fasciculus integrity. J Int Neuropsychorogical Soc. (2011) 17:663–73. 10.1017/S1355617711000464 21492497 PMC3707392

[63] Hirstein W Ramachandran VS. Capgras syndrome: a novel probe for understanding the neural representation of the identitiy and familiarity of persons. Proc Biol Sci. (1997) 264:437–44. 10.1098/rspb.1997.0062 9107057 PMC1688258

[64] Edelstyn NMJ Oyebode F Barrett K. The delusions of Capgras and intermetamorphosis in a patient with right-hemisphere white-matter pathology. Psychopathology (2001) 34:299–304. 10.1159/000049328 11847489

[65] Hennig-Fast K Michl P Müller J Niedermeier N Coates U Müller N. Obsessive-compulsive disorder - A question of conscience? An fMRI study of behavioural and neurofunctional correlates of shame and guilt. J Psychiatr Res. (2015) 68:354–62. 10.1016/j.jpsychires.2015.05.001 26028547

[66] Admon R Bleich-Cohen M Weizmant R Poyurovsky M Faragian S Hendler T. Functional and structural neural indices of risk aversion in obsessive-compulsive disorder (OCD). Psychiatry Res Neuroimaging (2012) 203:207–13. 10.1016/j.pscychresns.2012.02.002 22959813

[67] Benedetti F Giacosa C Radaelli D Poletti S Pozzi E Dallaspezia S. Widespread changes of white matter microstructure in obsessive-compulsive disorder: effect of drug status. Eur Neuropsychopharmacol (2013) 23:581–93. 10.1016/j.euroneuro.2012.07.002 22954900

[68] Jayarajan RN Venkatasubramanian G Viswanath B Reddy YCJ Srinath S Vasudev MK. White matter abnormalities in children and adolescents with obsessive-compulsive disorder: a diffusion tensor imaging study. Depress Anxiety (2012) 29:780–8. 10.1002/da.21890 22323419

[69] Zarei M Mataix-Cols D Heyman I Hough M Doherty J Burge L. Changes in gray matter volume and white matter microstructure in adolescents with obsessive-compulsive disorder. Biol Psychiatry (2011) 70:1083–90. 10.1016/j.biopsych.2011.06.032 21903200

[70] Molnar-Szakacs I Wang MJ Laugeson EA Overy K Wu WL Piggot J. Autism, emotion recognition and the mirror neuron system: the case of music. Mcgill J Med. (2009) 12:87–98. 21264050 PMC2997252

[71] Gotts SJ Simmons WK Milbury LA Wallace GL Cox RW Martin A. Fractionation of social brain circuits in autism spectrum disorders. Brain (2012) 135:2711–25. 10.1093/brain/aws160 22791801 PMC3437021

[72] Elison JT Wolff JJ Heimer DC Paterson SJ Gu H Hazlett HC. Frontolimbic neural circuitry at 6 months predicts individual differences in joint attention at 9 months. Dev Sci. (2013) 16:186–97. 10.1111/desc.12015 23432829 PMC3582040

[73] Kumar A Sundaram SK Sivaswamy L Behen ME Makki MI Ager J. Alterations in frontal lobe tracts and corpus callosum in young children with autism spectrum disorder. Cereb Cortex. (2010) 20:2103–13. 10.1093/cercor/bhp278 20019145

[74] Pardini M Elia M Garaci FG. Long-term cognitive and behavioral therapies, combined with augmentative communication, are related to uncinate fasciculus integrity in autism. J Autism Dev Disord. (2012) 42:585–92. 10.1007/s10803-011-1281-2 21573693

[75] Samson AC Dougherty RF Lee IA Phillips JM Gross JJ Hardan AY. Psychiatry research : neuroimaging white matter structure in the uncinate fasciculus : Implications for socio-affective deficits in Autism Spectrum Disorder. Psychiatry Res Neuroimaging (2016) 255:66–74. 10.1016/j.pscychresns.2016.08.004 27552717

[76] Yoo SY Jang JH Shin YW Kim DJ Park HJ Moon WJ. White matter abnormalities in drug-naïve patients with obsessive-compulsive disorder: a Diffusion Tensor Study before and after citalopram treatment. Acta Psychiatr Scand. (2007) 116:211–9. 10.1111/j.1600-0447.2007.01046.x 17655563

[77] Sexton CE Mackay CE Ebmeier KP. A systematic review of diffusion tensor imaging studies in affective disorders. Biol Psychiatry (2009) 66:814–23. 10.1016/j.biopsych.2009.05.024 19615671

[78] Solders SK Carper RA Müller RA. White matter compromise in autism? Differentiating motion confounds from true differences in diffusion tensor imaging. Autism Res. (2017) 10:1606–20. 10.1002/aur.1807 28503904 PMC5648623

